# COSMIN Risk of Bias checklist for systematic reviews of Patient-Reported Outcome Measures

**DOI:** 10.1007/s11136-017-1765-4

**Published:** 2017-12-19

**Authors:** L. B. Mokkink, H. C. W. de Vet, C. A. C. Prinsen, D. L. Patrick, J. Alonso, L. M. Bouter, C. B. Terwee

**Affiliations:** 1 Department of Epidemiology and Biostatistics and Amsterdam Public Health Research Institute, VU University Medical Center, P.O. Box 7057, 1007 Amsterdam, The Netherlands; 2 Department of Health Services, University of Washington, Seattle, WA USA; 3 Health Services Research Unit, Institut Municipal d’Investigacio Medica (IMIM-Hospital del Mar), Barcelona, Spain; 4 Department of Philosophy, Faculty of Humanities, Vrije Universiteit, Amsterdam, The Netherlands

**Keywords:** Quality assessment, Systematic review, Risk of bias, Measurement properties, Outcome measurement instruments

## Abstract

**Purpose:**

The original COnsensus-based Standards for the selection of health Measurement INstruments (COSMIN) checklist was developed to assess the methodological quality of single studies on measurement properties of Patient-Reported Outcome Measures (PROMs). Now it is our aim to adapt the COSMIN checklist and its four-point rating system into a version exclusively for use in systematic reviews of PROMs, aiming to assess risk of bias of studies on measurement properties.

**Methods:**

For each standard (i.e., a design requirement or preferred statistical method), it was discussed within the COSMIN steering committee if and how it should be adapted. The adapted checklist was pilot-tested to strengthen content validity in a systematic review on the quality of PROMs for patients with hand osteoarthritis.

**Results:**

Most important changes were the reordering of the measurement properties to be assessed in a systematic review of PROMs; the deletion of standards that concerned reporting issues and standards that not necessarily lead to biased results; the integration of standards on general requirements for studies on item response theory with standards for specific measurement properties; the recommendation to the review team to specify hypotheses for construct validity and responsiveness in advance, and subsequently the removal of the standards about formulating hypotheses; and the change in the labels of the four-point rating system.

**Conclusions:**

The COSMIN Risk of Bias checklist was developed exclusively for use in systematic reviews of PROMs to distinguish this application from other purposes of assessing the methodological quality of studies on measurement properties, such as guidance for designing or reporting a study on the measurement properties.

**Electronic supplementary material:**

The online version of this article (10.1007/s11136-017-1765-4) contains supplementary material, which is available to authorized users.

## Background

Research performed with Patient-Reported Outcome Measures (PROMs) of poor or unknown quality constitutes a waste of resources and is unethical [1]. Unfortunately this practice is widespread [2]. Selecting the best PROM for the outcome of interest (i.e., to be used in an evaluative application) in a methodologically sound way requires (1) high-quality studies on the measurement properties of relevant PROMs in the target population and (2) a high-quality systematic review of studies on measurement properties in which all information is gathered and evaluated in a systematic and transparent way.

In a systematic review of PROMs, the methodological quality of included studies and the quality of the PROMs (i.e., its measurement properties) should be assessed separately. Based on all information, the quality of evidence can be graded [3]. Assessing the methodological quality of studies is important, because the quality of the study determines the trustworthiness of the results (i.e., risk of bias).

The COnsensus-based Standards for the selection of health Measurement INstruments (COSMIN) checklist is a tool developed to assess the methodological quality of single studies on measurement properties of PROMs [4, 5]; likewise, the Quality Assessment of Diagnostic Accuracy Studies (QUADAS) tool is for diagnostic studies [6]. It contains standards referring to design requirements and preferred statistical methods of studies on measurement properties. For example, internal consistency should be assessed for each unidimensional scale or subscale separately (design requirement), and Cronbach’s alpha is the preferred statistical method. For each measurement property, a COSMIN box was developed containing all standards needed to assess the quality of a study on that specific measurement property. The methodological quality of each single study included in a review is evaluated by rating all standards included in the accompanying box. For each study, an overall judgement is needed on the quality of the particular study. We therefore developed a four-point rating system where each standard within a COSMIN box can be rated as ‘excellent,’ ‘good,’ ‘fair,’ or ‘poor’ [7]. The overall rating of the quality of each study is determined by taking the lowest rating of any standard in the box (i.e., the ‘worst score counts’ principle) [7]. This overall rating of the quality of the studies is taken into account when grading the quality of the evidence on the measurement property of a PROM [3].

The number of systematic reviews of PROMs published is increasing, and the COSMIN checklist is widely used in many of these reviews. A decade after its publication, an update seemed necessary. Users of the COSMIN checklist, including us, have suggested several improvements to the checklist—published and unpublished. For example, a study often received a ‘fair’ quality rating only because it was not reported how missing items were handled. It was argued that this would not necessarily lead to biased results of the study. Or a well-performed study was rated as ‘poor’ only because the sample size was small. It was argued that a number of small high-quality studies together can provide good evidence for the measurement properties of an instrument if the results are pooled. Furthermore, users found it somewhat confusing how to handle studies that use item response theory (IRT) methods. It was unclear how to use the box General requirements for IRT studies in combination with the IRT standards for measurement properties.

The methodological quality of studies can be assessed for different purposes, for example, as guidance for designing or reporting a study on the measurement properties, to determine the risk of bias of results of single studies included in a systematic review of PROMs, or by reviewers or journal editors to appraise the methodological quality of articles or grant applications of studies on measurement properties [5]. The COSMIN steering committee (i.e., all authors) agreed that it would be desirable to develop different versions of the COSMIN checklist for these different purposes as the standards included in each checklist will be slightly different. For example, when designing a study, it might be useful to have a standard on the preferred sample size, and when reporting a study on measurement properties, it is useful to have a standard on reporting missing data. Therefore, three versions of the COSMIN checklist were proposed: (1) COSMIN Study Design checklist (available from our website http://www.cosmin.nl), (2) COSMIN Risk of Bias checklist (at issue in this article), and (3) COSMIN Reporting checklist (PCORI reference: 1606–35,556, ongoing research).

In this paper, we describe and elaborate on the changes that were made in each standard, with the exception of the standards from the box for content validity. This box was recently adapted based on an international Delphi study. The results of this study are described elsewhere [8]. The response options for the four-point rating system were also adapted. The standards of all measurement properties and the four-point rating system together form the COSMIN Risk of Bias checklist. To determine risk of bias in a study on measurement properties, the methodological quality of a study is assessed using the ‘worst score counts’ principle [7]. Details on how to use the COSMIN Risk of Bias checklist in a systematic review of PROMs and how to grade the evidence, taking the methodological quality of the studies into account, are described elsewhere [3, 9].

## Methods

The term ‘risk of bias’ is in compliance with the Cochrane methodology for systematic reviews of trials and diagnostic studies [10]. It refers to whether you can trust results based on the methodological quality of the study.

Each standard and response category was critically reviewed and necessary changes were made. We aimed to keep only those standards that concerned risk of bias of study results, and therefore standards that exclusively concerned reporting issues were deleted. In addition, the order of the boxes was changed to reflect the suggested order of evaluating measurement properties in a systematic review of PROMs [3]. Comments and suggestions for improvement were collected from 41 reviews included in the COSMIN database of systematic reviews that had used the COSMIN checklist. Decisions on adaptations were made based on iterative discussions by the COSMIN steering committee, both at face-to-face meetings (LM, CP, HdV, and CT) and by email discussions (entire COSMIN steering committee). All members of the steering committee have experience in PROM development and testing, qualitative and quantitative research on assessing measurement properties, and systematic reviews of PROMs.

The adapted checklist was pilot-tested by four authors (LM, HdV, CP, and CT) by rating the risk of bias of the studies on measurement properties described in eight articles included in a systematic review of PROMs for patients with hand osteoarthritis (manuscript in preparation). The aim of the pilot test was to strengthen content validity of the COSMIN Risk of Bias checklist. Proposals for small modifications in wording and rating of the standards after the pilot test were discussed and approved within the steering committee.

## Results

### New ordering of the boxes of the measurement properties

In the guideline for a systematic review of PROMs, a new order of evaluating the measurement properties is proposed [3]. Therefore, the ordering of the boxes in the COSMIN Risk of Bias checklist was accordingly changed. This new ordering is shown in Table 1. Boxes 1 and 2 address content validity. Content validity is considered to be the most important measurement property because first of all it should be clear that the items of the PROM are relevant, comprehensive, and comprehensible with respect to the construct of interest and target population [3].

**Table 1:** Boxes in the original COSMIN checklist (left) and the COSMIN Risk of Bias checklist (right)

Boxes of the original COSMIN checklist	Boxes of the COSMIN Risk of Bias checklist
Box General requirements for studies that applied IRT models	*Content validity*
Box A. Internal consistency	Box 1. PROM development
Box B. Reliability	Box 2. Content validity
Box C. Measurement error	*Internal structure*
Box D. Content validity (including face validity)	Box 3. Structural validity
Box E. Structural validity	Box 4. Internal consistency
Box F. Hypotheses testing	Box 5. Cross-cultural validity\measurement invariance
Box G. Cross-cultural validity	*Remaining measurement properties*
Box H. Criterion validity	Box 6. Reliability
Box I. Responsiveness	Box 7. Measurement error
Box J. Interpretability	Box 8. Criterion validity
Box Generalisability	Box 9. Hypotheses testing for construct validity
	Box 10. Responsiveness

Boxes 3–5 address structural validity, internal consistency, and cross-cultural validity\measurement invariance, respectively, together reflecting the internal structure of the PROM. Internal structure refers to how the different items in a PROM are related, which is important to know for deciding how items might be combined into scales or subscales. Evaluating the internal structure of the instrument is relevant for PROMs that are based on a reflective model. In a reflective model, the construct manifests itself in the items, i.e., the items are a reflection of the construct to be measured [11]. This step concerns an evaluation of (a) structural validity (including unidimensionality) using factor analyses or IRT or Rasch analyses, (b) internal consistency, and (c) cross-cultural validity and other forms of measurement invariance (MI) [using Differential Item Functioning (DIF) analyses or Multi-Group Confirmatory Factor Analyses (MGCFA)]. These three measurement properties focus on the quality of items and the relationships between items. It is recommended to evaluate these measurement properties immediately after evaluating the content validity of a PROM. As evidence for the unidimensionality or structural validity of a scale or subscale is a prerequisite for the interpretation of internal consistency analyses (e.g., Cronbach’s alphas), it is recommended to first evaluate structural validity, to be followed by internal consistency.

We recommend to evaluate cross-cultural validity for PROMs that are used in culturally different populations than originally developed for. We interpret ‘culturally different population’ broadly. We do not only consider different ethnicity or language groups as different cultural populations, but also other groups such as different gender or age groups, or different patient populations. Cross-cultural validity is evaluated by assessing whether the scale is measurement invariant or whether DIF occurs. MI and non-DIF refer to whether respondents from different groups with the same latent trait level (allowing for group differences) respond similarly to a particular item. The term MI is an overarching term. However, we decided not to delete terms from the COSMIN taxonomy. Therefore, the box is now called cross-cultural validity\measurement invariance.

The boxes 6–10 address the remaining measurement properties (i.e., reliability, measurement error, criterion validity, hypotheses testing for construct validity, and responsiveness). We do not consider one of these measurement properties as more important than the others. These measurement properties mainly focus on the quality of the (sub)scale as a whole, rather than on item level.

### Removal of boxes

The boxes General requirements for studies that applied IRT models, Interpretability, and Generalizability have been removed from the checklist.

We removed the box General requirements for IRT or Rasch analyses. The first three standards of this box concerned reporting of the IRT model, the computer software package, and the method of estimation. These reporting items do not concern the quality of the studies in terms of risk of bias. The fourth item concerned whether IRT assumptions like unidimensionality and local independence were checked. These issues are removed because lack of testing for these assumptions does not necessarily indicate poor quality of the study. That is, if the model fits, unidimensionality and local dependence can be assumed and do not need to be checked. But when a poor IRT or Rasch model fit is found, one may examine if unmet assumptions can explain the misfit of the model. Furthermore, the quality of studies on unidimensionality of scales or subscales is considered in the box Structural validity. The fourth item also concerned whether DIF analyses were performed. We have moved this standard to the box Cross-cultural validity\measurement invariance, as it tests whether items behave similarly in different groups. Standards on preferred statistical methods based on IRT or Rasch analyses are included in the boxes Internal consistency, Structural validity, and Cross-cultural validity\measurement invariance, similarly as in the original COSMIN checklist.

The box Interpretability contained items referring to the reporting of information to facilitate interpretation of (change) scores, rather than standards to assess risk of bias of a study on interpretability. Moreover, interpretability is not a measurement property. Despite its importance, it was decided to remove this box because the checklist focuses on risk of bias.

The box Generalizability contained items on whether the study population is adequately described in terms of age, gender, and important disease characteristics. These items also do not refer to risk of bias and were therefore removed.

### Adaptations of individual standards

Table 2 provides an overview of the adaptations resulting in the COSMIN Risk of Bias checklist.

**Table 2:** Overview of changes in COSMIN standards

Standard^a^	Short description of standard	Description of change
IRT 1	IRT model	Deleted, reporting issue
IRT 2	Software package	Deleted, reporting issue
IRT 3	Method of estimation	Deleted, reporting issue
IRT 4	Assumptions and item fit	Assumptions deleted, reporting issue; item fit/DIF moved to the box Cross-cultural validity\measurement invariance
A1	Effect indicators	Not RoB/standard, included for reviewer’s information
A2	% Missing item	Deleted, not RoB
A3	Handling missings	Deleted, not RoB
A4	Sample size	Deleted, not RoB
A5	Check unidimensionality	Deleted, redundant due to new order
A6	Sample size	Deleted, redundant due to new order
B1	% Missing item	Deleted, not RoB
B2	Handling missings	Deleted, not RoB
B3	Sample size	Deleted, not RoB
B4	Two measurements available	Not RoB
B5	Independent administrations	Deleted, does not discriminate between studies^b^
B6	Time interval stated	Deleted, not RoB
C1	% Missing item	Deleted, not RoB
C2	Handling missings	Deleted, not RoB
C3	Sample size	Deleted, not RoB
C4	Two measurements available	Deleted, not RoB
C5	Independent administrations	Deleted, does not discriminate between studies^b^
C6	Time interval stated	Deleted, not RoB
	Percentage agreement	Removed from poor-reliability method to excellent measurement error method
E1	Effect indicators	Not RoB/standard, but included for reviewer’s information
E2	% Missing item	Deleted, not RoB
E3	Handling missings	Deleted, not RoB
E4	Sample size	Four-point rating scale adapted
F1	% Missing item	Deleted, not RoB
F2	Handling missings	Deleted, not RoB
F3	Sample size	Deleted, not RoB
F4	Hypotheses formulated	Deleted, not RoB
F5	Direction	Deleted, not RoB
F6	Magnitude	Deleted, not RoB
F7	Description of comparator instrument	Formulation adapted
F8	Description of measurement properties	Formulation adapted
	Description characteristics of subgroups	Newly added
G1	% Missing item	Deleted, not RoB
G2	Handling missings	Deleted, not RoB
G3	Sample size	Four-point rating scale adapted
G4-G11	Translation process	Deleted, not RoB
H1	% Missing item	Deleted, not RoB
H2	Handling missings	Deleted, not RoB
H3	Sample size	Deleted, not RoB
I1	% Missing item	Deleted, not RoB
I2	Handling missings	Deleted, not RoB
I3	Sample size	Deleted, not RoB
I4	Longitudinal design	Deleted, not RoB
I5	Time interval stated	Deleted, not RoB
I6	Anything occurred	Deleted, not RoB
I7	Proportion patients changed	Deleted, not RoB
I8	Hypotheses formulated	Deleted, not RoB
I9	Direction	Deleted, not RoB
I10	Magnitude	Deleted, not RoB
I11	Description of comparator instrument	Formulation adapted
I12	Description of measurement properties	Formulation adapted
	Description characteristics of subgroups	Newly added

*RoB* risk of bias, *DIF* differential item functioning

^a^Standard number refers to original numbering of items [5]

^b^Usually all studies scored excellent on this item

#### Removal of standards on missing data and handling missing data

In the original COSMIN checklist, each box, except for the content validity box, contained standards about whether the percentage missing items was reported, and how these missing items were handled. Although we consider information on missing items very important to report, we decided to remove these standards from all boxes, as it was agreed that lack of reporting on the number of missing items and on how missing items were handled would not necessarily lead to biased results of the study. Furthermore, at the moment there is little evidence about what the best way is to handle missing items in studies on measurement properties.

#### Removal of standards on sample size

We decided to remove the standard about adequate sample size for single studies from those boxes where it is possible to pool the results (i.e., the boxes Internal consistency, Reliability, Measurement error, Criterion validity, Hypotheses testing for construct validity, and Responsiveness) to a later phase of the review, i.e., when drawing conclusions across studies on the measurement properties of the PROM [3]. This was decided because several small high-quality studies can together provide good evidence for the measurement property. Therefore, we recommend to take the aggregated sample size of the available studies into account when assessing the overall quality of evidence on a measurement property in a systematic review, as is described in detail elsewhere [3]. This is in compliance with Cochrane guidelines [10]. However, the standard about adequate sample size for single studies was maintained in the boxes Structural validity and Cross-cultural validity\measurement invariance, because the results of these studies cannot be pooled. In these boxes, factor analyses, or IRT or Rasch analyses, are included as preferred statistical methods and these methods require sample sizes that are sufficiently large to obtain reliable results.

The suggested sample size requirements should be considered as the basic rules; in some situations, dependent on the type of model, number of factors or items, more nuanced criteria might be applied. For example, a smaller sample size might be acceptable when an argument is presented in the individual study, stating the considerations why a smaller sample size is adequate. Subsequently, the study can still be rated as very good or adequate, despite lower sizes than requested in the standard.

Detailed information is provided in the ‘COSMIN methodology for systematic reviews of Patient-Reported Outcome Measures (PROMs)—user manual’ [9].

#### Removal of standards to determine which measurement property was assessed

In the original COSMIN checklist, several standards were included to determine whether or not a specific measurement property was evaluated. For example, in the boxes Reliability and Measurement error, it was asked whether at least two measurements were available; in the boxes Internal consistency and Structural validity, a standard was included whether the scale consists of effect indicators. If the answer was ‘no,’ the measurement property was not relevant. These questions do not refer to the risk of bias of the study, but to the relevance of the study, and are therefore no longer considered as standards. They are now either deleted or the item number was removed (to indicate that it is not a standard) and instructions were added (i.e., ‘if no, the study can be ignored’).

#### Removal of redundant standards from the box Internal consistency

Unidimensionality is a prerequisite for a proper interpretation of the internal consistency statistic. In the original version of the COSMIN checklist, two standards were included in the box Internal consistency about checking this assumption, i.e., “Was the unidimensionality checked, i.e., was factor analysis or IRT model applied?” and “Was the sample size included in the unidimensionality analysis adequate?” We have removed these items from the box, because according to the new order of evaluating measurement properties it should first be checked whether there is evidence that a scale or subscale is unidimensional, using the box Structural validity, before evaluating internal consistency.

#### Removal of standards about the translation process

In the original COSMIN checklist, the box Cross-cultural validity included both standards for assessing the quality of the translation process and standards for assessing the quality of a cross-cultural validity study. We decided to remove the standards for assessing the quality of the translation process because the translation process itself is not a measurement property; performing a pilot test after a translation is considered part of content validity (i.e., an evaluation of comprehensibility) and now included in the box Content validity [8], and a poor translation process does not necessarily mean that the instrument has a poor cross-cultural validity.

#### Changes in the boxes Criterion validity, Hypotheses testing for construct validity, and Responsiveness

We decided to delete the standard about a reasonable gold standard and all standards about formulating hypotheses a priori from these boxes. We consider it important to determine whether a ‘gold standard’ can indeed be considered a ‘gold standard.’ However, when conducting a systematic review of PROMs, we now recommend that the review team determines before assessing the quality of included studies which outcome measurement instruments can indeed be considered a ‘gold standard.’ Next, although we consider it majorly important to define hypotheses in advance when assessing construct validity or responsiveness of a PROM, results of studies without these hypotheses can in many cases still be used in a systematic review on PROMs because the presented correlations or mean differences between (sub)groups are not necessarily biased. The conclusions of the authors though are often biased when a priori hypotheses are lacking. We recommend that the review team formulates hypotheses themselves about the expected direction and magnitude of correlations between the PROM of interest and other PROMs and of mean differences in scores between groups [12], and compare the results found in the included studies to the hypotheses formulated by the review team. If construct validity studies do include hypotheses, the review team can adopt these hypotheses if they consider them adequate. This way, the results from many studies can still be used in the systematic review as studies without hypotheses will no longer receive an ‘inadequate’ (previously called ‘poor’) quality rating. An additional advantage of this approach is that the results of all included studies are compared to the same set of hypotheses. A detailed explanation for completing these boxes can be found in the manual of the checklist [9].

To improve the comprehensibility of the boxes Hypotheses testing for construct validity and Responsiveness, we now include separate sections for different study designs in these boxes. These sections concern standards for testing hypotheses about comparing (changes on) the outcome measurement instrument of interest with (changes on the) comparator outcome measurement instruments (e.g., convergent validity), or standards for comparing (changes in) scores between subgroups (discriminative or known-groups validity). We also included a separate section in the box Responsiveness containing standards for studies in which effect sizes and related parameters are being used. In the sections on comparison between subgroups, we added a standard whether an adequate description was provided of important characteristics of the subgroup.

Finally, several standards were reformulated to change them from a reporting standard into a standard for risk of bias assessment. For example, we changed the original standard “Was an adequate description provided of the comparator instrument(s)?” into “Is it clear what the comparator instrument(s) measure(s)?” This standard can be answered based on information from the article, but also based on additional information from the literature.

### New labels for the four-point rating system

It was argued that the original labels of the four-point rating scale (i.e., ‘excellent,’ ‘good,’ ‘fair,’ ‘poor’) do not appropriately reflect the judgments given, because the labels do not exactly match the descriptions used in the boxes. The descriptions of the category ‘fair’ often used the words doubtful and unclear. Therefore, a label ‘doubtful’ was considered more appropriate. The labels ‘good’ and ‘poor’ were not considered symmetrical and were therefore changed into ‘adequate’ and ‘inadequate.’ Lastly, we wanted to have a category to reflect studies that performed very well. We changed ‘excellent’ into ‘very good’ because we considered the latter to reflect the distance between the response categories more appropriately. Also, by changing all labels, the difference between the original and new COSMIN checklist would be more clear for users.

### Availability

The COSMIN Risk of Bias checklist for systematic reviews of PROMs is presented in the Appendix and on the COSMIN website. The ‘COSMIN methodology for systematic reviews of Patient-Reported Outcome Measures (PROMs)—user manual’ is also published on the COSMIN website [9] with detailed instructions about how each standard should be rated.

## Discussion

In this paper, we present the COSMIN Risk of Bias checklist for use in systematic reviews of PROMs to assess the methodological quality of single studies on measurement properties. The boxes PROM development and Content validity are discussed elsewhere [8]. The boxes of the other measurement properties were developed based on published and unpublished input and experiences from users of the original COSMIN checklist and iterative discussions among members of the COSMIN steering committee, and pilot-tested in an ongoing systematic review to strengthen content validity of the COSMIN Risk of Bias checklist. The COSMIN Risk of Bias checklist only contains standards that address the risk of bias of a study. We changed the order of the boxes of the checklist, reflecting the order of evaluating the measurement properties [3]. We removed all standards that concerned reporting issues and standards that do not necessarily lead to biased results, and therefore removed the boxes General requirements for studies that applied IRT models, Generalizability, and Interpretability. We integrated the standards on general requirements for studies on item response theory with standards for specific measurement properties. We now recommend the review team to specify hypotheses for construct validity and responsiveness in advance and subsequently removed the standards about formulating hypotheses. Last, changes in the labeling of the four-point rating system were made. All these changes have rendered a more adequate and specific checklist for assessing the risk of bias of study results in systematic reviews of PROMs.

To determine the quality of a PROM, a systematic approach is deemed necessary, in which all evidence on the measurement properties of the PROM is considered, taking into account the risk of bias of the studies in which the measurement properties were determined. COSMIN has developed a methodology for systematic reviews of PROMs in which such a systematic approach is described [3]. In concordance with existing guidelines for other types of systematic reviews, we distinguish several steps, including systematically searching and selecting relevant articles, evaluating the quality of the eligible studies, evaluating the measurement properties of the included instruments, evaluating interpretability and feasibility aspects, formulating conclusions and recommendations, and publishing [3]. The COSMIN Risk of Bias checklist can be used for one of these steps, that is, to evaluate the risk of bias of eligible studies. Other COSMIN tools were developed for other steps, such as a search filter for finding studies on measurement properties [13] and content validity methodology [8]. Based on the GRADE approach, we developed a method to systematically draw conclusions per measurement property on the quality of a PROM [3].

Apart from the COSMIN methodology to assess the quality of PROMs, other guidelines exist, such as the standards of the American Psychological Association (APA), the attributes and criteria of the Scientific Advisory Committee of the Medical Outcome Trust (SAC-MOS) [14], the Evaluating the Measurement of Patient-Reported Outcomes (EMPRO) tool [15] (based on SAC-MOS), the Terwee criteria [16], and a recently published checklist by Francis et al. [17]. The majority of these tools are much shorter, but these guidelines do not fully describe and explain all steps needed to determine the quality of a PROM in a systematic way. For example, in these guidelines it is not stated that the conclusion should be based on a systematic consideration of all existing evidence. Many of these guidelines combine standards for the quality of the studies with criteria for rating the results of the measurement properties into one standard. For example, one of the EMPRO standards on validity states that “the hypotheses regarding construct validity are specifically described and the results are consistent with them,” which addressed the quality of the study (specific hypotheses) and the quality of the instrument (results consistent with hypotheses) at the same time. Next, in contrast to the COSMIN methodology, these guidelines do not describe how the results from different studies with different quality should be combined to draw an overall conclusion on a PROM. This is also not described in the Terwee criteria [16] or the checklist of Francis et al. [17]. Furthermore, these standards are not detailed enough to provide transparent and systematic ratings of the risk of bias of studies on the measurement properties. For example, Cronbach’s alpha can only be interpreted as a measure of internal consistency when the scale or subscale is unidimensional [18]. When the assumption of unidimensionality is not met, the Cronbach’s alpha may overestimate the true internal consistency. However, in some guidelines a requirement to assess unidimensionality (e.g., by factor analysis) is not included. For example, both the EMPRO standards [15] and the standards by Francis et al. [17] on internal consistency do not explicitly address whether the scale or subscale was unidimensional. To assess construct validity, Francis et al.’s checklist [17] only asks about whether “there are findings supporting existing associations with existing PRO measures or with other relevant data,” without requiring any information on the quality of those existing PROMs. Also, some items refer to reporting issues (i.e., whether methods are clearly described) or generalizability (i.e., characteristics of the sample) of the findings. These issues are not likely to lead to bias of results. A last issue is that some measurement properties are not specifically mentioned. For example, several guidelines do not distinguish between reliability and measurement error [14, 15, 17]. The simplicity and lack of detail of these guidelines may limit their practical application by reviewers if they are not experts in PROM development and measurement properties. To improve the transparency and quality of systematic reviews on PROMs, we recommend to use the COSMIN methodology, including the COSMIN Risk of Bias checklist.

There are some limitations to our study. Due to shortage on resources, we did not perform a Delphi study to develop the COSMIN Risk of Bias checklist, nor collected all criticism on the original COSMIN checklist in a systematic way. We did not prospectively contact users of the COSMIN checklist to ask for suggestions for improvement.

We believe that the COSMIN Risk of Bias checklist will lead to a better assessment of whether results found in studies on the measurement properties of PROMs can be trusted and used in a systematic review of PROMs than the assessment based on the original COSMIN checklist. By developing different versions of the checklist designed for its specific purpose, i.e., the COSMIN Study Design checklist, the COSMIN Risk of Bias checklist, and the upcoming COSMIN Reporting checklist, we believe that we better serve users who want to assess the methodological quality of studies on measurement properties for different reasons.

## Electronic supplementary material

Below is the link to the electronic supplementary material.

Supplementary material 1 (DOC 470 KB)

## Abbreviations

COSMIN: COnsensus-based Standards for the selection of health Measurement INstruments
PROM: Patient-Reported Outcome Measures
IRT: Item response theory
DIF: Differential item functioning
MI: Measurement invariance
MIC: Minimal important change
MGCFA: Multi-group confirmatory factor analyses
RoB: Risk of bias
