# Supplementary material for: COSMIN Risk of Bias checklist for systematic reviews of Patient-Reported Outcome Measures
Source: Qual Life Res. 2017 Dec 19;27(5):1171–9. doi: 10.1007/s11136-017-1765-4 (PMC5891552; doi:10.1007/s11136-017-1765-4)
Supplement: Supplementary file 1 — Supplementary material 1 (DOC 470 KB) [file 11136_2017_1765_MOESM1_ESM.doc]

**COSMIN Risk of Bias checklist**

**Date:** December, 2017

**Contact**

L.B. Mokkink, PhD

VU University Medical Center

Department of Epidemiology and Biostatistics

Amsterdam Public Health research institute

P.O. box 7057

1007 MB Amsterdam

The Netherlands

Website: [www.cosmin.nl](http://www.cosmin.nl/)

E-mail: [w.mokkink@vumc.nl](mailto:w.mokkink@vumc.nl)

*How to site the COSMIN Risk of Bias Checklist*

Please refer to the following studies when using the COSMIN Risk of Bias Checklist:

Mokkink, L.B., De Vet, H.C.W., Prinsen, C.A.C, Patrick, D.L., Alonso, J., Bouter, L.M., et al. COSMIN Risk of Bias checklist for systematic reviews of Patient-Reported Outcome Measures. Accepted for publication in Quality of Life Research.

Prinsen, C. A., Mokkink, L. B., Bouter, L. M., Alonso, J., Patrick, D. L., Vet, H. C., et al. COSMIN guideline for systematic reviews of Patient-Reported Outcome Measures. Submitted.

Terwee, C. B., Prinsen, C. A., Chiarotto, A., Vet, H. C., Westerman, M. J., Patrick, D. L., et al. COSMIN methodology for evaluating the content validity of Patient-Reported Outcome Measures: a Delphi study. Submitted.

For details on how to use the COSMIN risk of Bias checklist see ‘COSMIN methodology for systematic reviews of Patient-Reported Outcome Measures (PROMs) – user manual’ and ‘COSMIN methodology for assessing the content validity of Patient-Reported Outcome Measures (PROMs) - user manual’ available from our website www.cosmin.nl.

*Abbreviations used:*

*CTT – classical test theory*

*DIF – differential item functioning*

*IRT – Item response theory*

*MGCFA – multi-group confirmatory factor analysis*

*MI – measurement invariance*

*NA – not applicable*

*PROM – patient-reported outcome measure*

*1PL model – 1 parameter IRT model*

*2PL model – 2 parameter IRT model*

**Instructions**

Tick the boxes that need to be completed for the article

|  | **COSMIN Risk of Bias checklist** |
| --- | --- |
|  | Box 1. PROM development |
|  | Box 2. Content validity |
|  | Box 3. Structural validity |
|  | Box 4. Internal consistency |
|  | Box 5. Cross-cultural validity\Measurement invariance |
|  | Box 6. Reliability |
|  | Box 7. Measurement error |
|  | Box 8. Criterion validity |
|  | Box 9. Hypotheses testing for construct validity |
|  | Box 10. Responsiveness |

To assess the methodological quality of each study, i.e. assessing the risk of bias of the result of a study, the corresponding COSMIN Risk of Bias box should be completed. To determine the overall quality of a study the lowest rating of any standard in the box is taken (i.e. “the worst score counts” principle). For example, if for a reliability study one item in a box is rated as ‘inadequate’, the overall methodological quality of that reliability study is rated as ‘inadequate’. The response option ‘NA’ (not applicable) is at issue for some standards. For example, when a study on structural validity is based on CTT, the standard on IRT is not applicable and this standard should not be considered in the “worst score counts”- rating for that specific study. For standards where this option is not at issue, these cells are grey and shouldn’t be used.

| **Box 1. PROM development** | | | | | | | |
| --- | --- | --- | --- | --- | --- | --- | --- |
| **1a. PROM design** | | |  |  |  |  |  |
| *General design requirements* | | | **very good** | **adequate** | **doubtful** | **inadequate** | **NA** |
|  |  | |  |  |  |  |  |
| 1 | | Is a clear description provided of the construct to be measured? | Construct clearly described |  |  | Construct not clearly described |  |
| 2 | | Is the origin of the construct clear: was a theory, conceptual framework or disease model used or clear rationale provided to define the construct to be measured? | Origin of the construct clear |  | Origin of the construct not clear |  |  |
| 3 | | Is a clear description provided of the target population for which the PROM was developed? | Target population clearly described |  |  | Target population not clearly described |  |
| 4 | | Is a clear description provided of the context of use | Context of use clearly described |  | Context of use not clearly described |  |  |
| 5 | | Was the PROM development study performed in a sample representing the target population for which the PROM was developed? | Study performed in a sample representing the target population | Assumable that the study was performed in a sample representing the target population, but not clearly described | Doubtful whether the study was performed in a sample representing the target population | Study not performed in a sample representing the target population  **(SKIP items 6-12)** |  |

| *Concept elicitation (relevance and comprehensiveness)* | | | **very good** | **adequate** | **doubtful** | **inadequate** | **NA** |
| --- | --- | --- | --- | --- | --- | --- | --- |
|  |  | |  |  |  |  |  |
| 6 | | Was an appropriate qualitative data collection method used to identify relevant items for a new PROM? | Widely recognized or well justified qualitative method used, suitable for the construct and study population | Assumable that the qualitative method was appropriate and suitable for the construct and study population, but not clearly described | Only quantitative (survey) method(s) used or doubtful whether the method was suitable for the construct and study population | Method used not appropriate or not suitable for the construct or study population |  |
| 7 | | Were skilled group moderators/interviewers used? | Skilled group moderators/ interviewers used | Group moderators /interviewers had limited experience or were trained specifically for the study | Not clear if group moderators /interviewers were trained or group moderators /interviewers not trained and no experience |  | Not applicable |
| 8 | | Were the group meetings or interviews based on an appropriate topic or interview guide? | Appropriate topic or interview guide | Assumable that the topic or interview guide was appropriate, but not clearly described | Not clear if a topic guide was used or doubtful if topic or interview guide was appropriate or no guide |  | Not applicable |
| 9 | | Were the group meetings or interviews recorded and transcribed verbatim? | All group meetings or interviews were recorded and transcribed verbatim | Assumable that all group meetings or interviews were recorded and transcribed verbatim, but not clearly described | Not clear if all group meetings of interviews were recorded and transcribed verbatim or recordings not transcribed verbatim or only notes were made during the group meetings/ interviews | No recording and no notes | Not applicable |
| 10 | | Was an appropriate approach used to analyse the data? | A widely recognized or well justified approach was used | Assumable that the approach was appropriate, but not clearly described | Not clear what approach was used or doubtful whether the approach was appropriate | Approach not appropriate |  |
| 11 | | Was at least part of the data coded independently? | At least 50% of the data was coded by at least two researchers independently | 11-49% of the data was coded by at least two researchers independently | Doubtful if two researchers were involved in the coding or only 1-10% of the data was coded by at least two researchers independently | Only one researcher was involved in coding or no coding | Not applicable |
| 12 | | Was data collection continued until saturation was reached? | Evidence provided that saturation was reached | Assumable that saturation was reached | Doubtful whether saturation was reached | Evidence suggests that saturation was not reached | Not applicable |
| 13 | | For quantitative studies (surveys): was the sample size appropriate? | ≥100 | 50-99 | 30-49 | <30 | Not applicable |

| **1b. Cognitive interview study or other pilot test** | | | | | | | |
| --- | --- | --- | --- | --- | --- | --- | --- |
|  |  | | **very good** | **adequate** | **doubtful** | **inadequate** | **NA** |
|  |  | |  |  |  |  |  |
| 14 | | Was a cognitive interview study or other pilot test conducted? | YES |  |  | **NO (SKIP items 15-35)** |  |
| *General design requirements* | | |  |  |  |  |  |
|  |  | |  |  |  |  |  |
| 15 | | Was the cognitive interview study or other pilot test performed in a sample representing the target population? | Study performed in a sample representing the target population | Assumable that the study was performed in a sample representing the target population, but not clearly described | Doubtful whether the study was performed in a sample representing the target population | Study not performed in a sample representing the target population |  |
| *Comprehensibility* | | |  |  |  |  |  |
|  |  | |  |  |  |  |  |
| 16 | | Were patients asked about the comprehensibility of the PROM? | YES |  | NO (SKIP items 17-25) | Not clear **(SKIP items 17-25)** |  |
| 17 | | Were all items tested in their final form? | All items were tested in their final form | Assumable that all items were tested in their final form, but not clearly described | Not clear if all items were tested in their final form | Items were not tested in their final form or items were not re-tested after substantial adjustments |  |
| 18 | | Was an appropriate qualitative method used to assess the comprehensibility of the PROM instructions, items, response options, and recall period? | Widely recognized or well justified qualitative method used | Assumable that the method was appropriate but not clearly described | Only quantitative (survey) method(s) used or doubtful whether the method was appropriate or not clear if patients were asked about the comprehensibility of the items, response options or recall period or patients not asked about the comprehensibility of the PROM instructions or the recall period | Method used not appropriate or patients not asked about the comprehensibility of the items or the response options |  |
| 19 | | Was each item tested in an appropriate number of patients?  For qualitative studies  For quantitative (survey) studies | ≥7  ≥50 | 4-6  ≥30 | <4 or not clear  <30 or not clear |  |  |
| 20 | | Were skilled interviewers used? | Skilled group moderators/ interviewers used | Group moderators /interviewers had limited experience or were trained specifically for the study | Not clear if group moderators /interviewers were trained or group moderators /interviewers not trained and no experience |  | Not applicable |
| 21 | | Were the interviews based on an appropriate interview guide? | Appropriate topic or interview guide | Assumable that the topic or interview guide was appropriate, but not clearly described | Not clear if a topic guide was used or doubtful if topic or interview guide was appropriate or no guide |  | Not applicable |
| 22 | | Were the interviews recorded and transcribed verbatim? | All group meetings or interviews were recorded and transcribed verbatim | Assumable that all group meetings or interviews were recorded and transcribed verbatim, but not clearly described | Not clear if all group meetings or interviews were recorded and transcribed verbatim or recordings not transcribed verbatim or only notes were made during the group meetings/ interviews | No recording and no notes | Not applicable |
| 23 | | Was an appropriate approach used to analyse the data? | A widely recognized or well justified approach was used | Assumable that the approach was appropriate, but not clearly described | Not clear what approach was used or doubtful whether the approach was appropriate | Approach not appropriate |  |
| 24 | | Were at least two researchers involved in the analysis? | At least two researchers involved in the analysis | Assumable that at least two researchers were involved in the analysis, but not clearly described | Not clear if two researchers were included in the analysis or only one researcher involved in the analysis |  |  |
| 25 | | Were problems regarding the comprehensibility of the PROM instructions, items, response options, and recall period appropriately addressed by adapting the PROM? | No problems found or problems appropriately addressed and PROM was adapted and re-tested if necessary | Assumable that there were no problems or that problems were appropriately addressed, but not clearly described | Not clear if there were problems or doubtful if problems were appropriately addressed | Problems not appropriately addressed or PROM was adapted but items were not re-tested after substantial adjustments. | Not applicable |

| *Comprehensiveness* | | | **very good** | **adequate** | **doubtful** | **inadequate** | **NA** | |
| --- | --- | --- | --- | --- | --- | --- | --- | --- |
|  |  | |  |  |  |  |  | |
| 26 | | Were patients asked about the comprehensiveness of the PROM? | YES |  | NO or not clear **(SKIP items 27-35)** |  |  | |
| 27 | | Was the final set of items tested? | The final set of items was tested | Assumable that the final set of items was tested, but not clearly described | Not clear if the final set of items was tested or not the final set of items was tested or the set of items was not re-tested after items were removed or added |  |  | |
| 28 | | Was an appropriate method used for assessing the comprehensiveness of the PROM? | Widely recognized or well justified method used | Assumable that the method was appropriate but not clearly described or only quantitative (survey) method(s) used | Doubtful whether the method was appropriate or method used not appropriate |  |  | |
| 29 | | Was each item tested in an appropriate number of patients?  For qualitative studies  For quantitative (survey) studies | ≥7  ≥50 | 4-6  ≥30 | <4 or not clear  <30 or not clear |  |  | |
| 30 | | Were skilled interviewers used? | Skilled interviewers used | Interviewers had limited experience or were trained specifically for the study | Not clear if interviewers were trained or interviewers not trained and no experience |  | | Not applicable |
| 31 | | Were the interviews based on an appropriate interview guide? | Appropriate topic or interview guide | Assumable that the topic or interview guide was appropriate, but not clearly described | Not clear if a topic guide was used or doubtful if topic or interview guide was appropriate or no guide |  | | Not applicable |
| 32 | | Were the interviews recorded and transcribed verbatim? | All group meetings or interviews were recorded and transcribed verbatim | Assumable that all group meetings or interviews were recorded and transcribed verbatim, but not clearly described | Not clear if all group meetings or interviews were recorded and transcribed verbatim or recordings not transcribed verbatim or only notes were made during the group meetings/ interviews or no recording and no notes |  | | Not applicable |
| 33 | | Was an appropriate approach used to analyse the data? | A widely recognized or well justified approach was used | Assumable that the approach was appropriate, but not clearly described | Not clear what approach was used or doubtful whether the approach was appropriate or approach not appropriate |  | |  |
| 34 | | Were at least two researchers involved in the analysis? | At least two researchers involved in the analysis | Assumable that at least two researchers were involved in the analysis, but not clearly described | Not clear if two researchers were included in the analysis or only one researcher involved in the analysis |  | |  |
| 35 | | Were problems regarding the comprehensiveness of the PROM appropriately addressed by adapting the PROM? | No problems found or problems appropriately addressed and PROM was adapted and re-tested if necessary | Assumable that there were no problems or that problems were appropriately addressed, but not clearly described | Not clear if there were problems or doubtful if problems were appropriately addressed or PROM was adapted but items were not re-tested after substantial adjustments | Problems not appropriately addressed | | Not applicable |

| **Box 2. Content validity** | | | | | | |
| --- | --- | --- | --- | --- | --- | --- |
| **2a. Asking patients about relevance** | | | | | | |
| *Design requirements* | | **very good** | **adequate** | **doubtful** | **inadequate** | **NA** |
|  | |  |  |  |  |  |
| 1 | Was an appropriate method used to ask patients whether each item is relevant for their experience with the condition? | Widely recognized or well justified method used | Only quantitative (survey) method(s) used or assumable that the method was appropriate but not clearly described | Not clear if patients were asked whether each item is relevant or doubtful whether the method was appropriate | Method used not appropriate or patients not asked about the relevance of all items |  |
| 2 | Was each item tested in an appropriate number of patients?  For qualitative studies  For quantitative (survey) studies | ≥7  ≥50 | 4-6  ≥30 | <4 or not clear  <30 or not clear |  |  |
| 3 | Were skilled group moderators/interviewers used? | Skilled group moderators/ interviewers used | Group moderators /interviewers had limited experience or were trained specifically for the study | Not clear if group moderators /interviewers were trained or group moderators /interviewers not trained and no experience |  | Not applicable |
| 4 | Were the group meetings or interviews based on an appropriate topic or interview guide? | Appropriate topic or interview guide | Assumable that the topic or interview guide was appropriate, but not clearly described | Not clear if a topic guide was used or doubtful if topic or interview guide was appropriate or no guide |  | Not applicable |
| 5 | Were the group meetings or interviews recorded and transcribed verbatim? | All group meetings or interviews were recorded and transcribed verbatim | Assumable that all group meetings or interviews were recorded and transcribed verbatim, but not clearly described | Not clear if all group meetings or interviews were recorded and transcribed verbatim or recordings not transcribed verbatim or only notes were made during the group meetings/ interviews | No recording and no notes | Not applicable |
| *Analyses* | |  |  |  |  |  |
|  |  |  |  |  |  |  |
| 6 | Was an appropriate approach used to analyse the data? | A widely recognized or well justified approach was used | Assumable that the approach was appropriate, but not clearly described | Not clear what approach was used or doubtful whether the approach was appropriate | Approach not appropriate |  |
| 7 | Were at least two researchers involved in the analysis? | At least two researchers involved in the analysis | Assumable that at least two researchers were involved in the analysis, but not clearly described | Not clear if two researchers were included in the analysis or only one researcher involved in the analysis |  |  |

| **2b Asking patients about comprehensiveness** | |  |  |  |  |  |
| --- | --- | --- | --- | --- | --- | --- |
| *Design requirements* | | **very good** | **adequate** | **doubtful** | **inadequate** | **NA** |
|  | |  |  |  |  |  |
| 8 | Was an appropriate method used for assessing the comprehensiveness of the PROM? | Widely recognized or well justified method used | Only quantitative (survey) method(s) used or assumable that the method was appropriate but not clearly described | Doubtful whether the method was appropriate | Method used not appropriate |  |
| 9 | Was each item tested in an appropriate number of patients?  For qualitative studies  For quantitative (survey) studies | ≥7  ≥50 | 4-6  ≥30 | <4 or not clear  <30 or not clear |  |  |
| 10 | Were skilled group moderators/interviewers used? | Skilled group moderators/ interviewers used | Group moderators /interviewers had limited experience or were trained specifically for the study | Not clear if group moderators /interviewers were trained or group moderators /interviewers not trained and no experience |  | Not applicable |
| 11 | Were the group meetings or interviews based on an appropriate topic or interview guide? | Appropriate topic or interview guide | Assumable that the topic or interview guide was appropriate, but not clearly described | Not clear if a topic guide was used or doubtful if topic or interview guide was appropriate or no guide |  | Not applicable |
| 12 | Were the group meetings or interviews recorded and transcribed verbatim? | All group meetings or interviews were recorded and transcribed verbatim | Assumable that all group meetings or interviews were recorded and transcribed verbatim, but not clearly described | Not clear if all group meetings or interviews were recorded and transcribed verbatim or recordings not transcribed verbatim or only notes were made during the group meetings/ interviews | No recording and no notes | Not applicable |
| *Analyses* | |  |  |  |  |  |
|  | |  |  |  |  |  |
| 13 | Was an appropriate approach used to analyse the data? | A widely recognized or well justified approach was used | Assumable that the approach was appropriate, but not clearly described | Not clear what approach was used or doubtful whether the approach was appropriate | Approach not appropriate |  |
| 14 | Were at least two researchers involved in the analysis? | At least two researchers involved in the analysis | Assumable that at least two researchers were involved in the analysis, but not clearly described | Not clear if two researchers were included in the analysis or only one researcher involved in the analysis |  |  |

| **2c Asking patients about comprehensibility** | | | | | | |
| --- | --- | --- | --- | --- | --- | --- |
| *Design requirements* | | **very good** | **adequate** | **doubtful** | **inadequate** | **NA** |
|  | |  |  |  |  |  |
| 15 | Was an appropriate qualitative method used for assessing the comprehensibility of the PROM instructions, items, response options, and recall period? | Widely recognized or well justified qualitative method used | Assumable that the method was appropriate but not clearly described | Only quantitative (survey) method(s) used or doubtful whether the method was appropriate or not clear if patients were asked about the comprehensibility of the items, response options or recall period or patients not asked about the comprehensibility of the PROM instructions | Method used not appropriate or patients not asked about the comprehensibility of the items, response options, or recall period |  |
| 16 | Was each item tested in an appropriate number of patients?  For qualitative studies  For quantitative (survey) studies | ≥7  ≥50 | 4-6  ≥30 | <4 or not clear  <30 or not clear |  |  |
| 17 | Were skilled group moderators/interviewers used? | Skilled group moderators/ interviewers used | Group moderators /interviewers had limited experience or were trained specifically for the study | Not clear if group moderators /interviewers were trained or group moderators /interviewers not trained and no experience |  |  |
| 18 | Were the group meetings or interviews based on an appropriate topic or interview guide? | Appropriate topic or interview guide | Assumable that the topic or interview guide was appropriate, but not clearly described | Not clear if a topic guide was used or doubtful if topic or interview guide was appropriate or no guide |  | Not applicable |
| 19 | Were the group meetings or interviews recorded and transcribed verbatim? | All group meetings or interviews were recorded and transcribed verbatim | Assumable that all group meetings or interviews were recorded and transcribed verbatim, but not clearly described | Not clear if all group meetings or interviews were recorded and transcribed verbatim or recordings not transcribed verbatim or only notes were made during the group meetings/ interviews | No recording and no notes | Not applicable |
| *Analyses* | |  |  |  |  |  |
|  | |  |  |  |  |  |
| 20 | Was an appropriate approach used to analyse the data? | A widely recognized or well justified approach was used | Assumable that the approach was appropriate, but not clearly described | Not clear what approach was used or doubtful whether the approach was appropriate | Approach not appropriate |  |
| 21 | Were at least two researchers involved in the analysis? | At least two researchers involved in the analysis | Assumable that at least two researchers were involved in the analysis, but not clearly described | Not clear if two researchers were included in the analysis or only one researcher involved in the analysis |  |  |

| **2d. Asking professionals about relevance** | |  |  |  |  |  |
| --- | --- | --- | --- | --- | --- | --- |
| *Design requirements* | | **very good** | **adequate** | **doubtful** | **inadequate** | **NA** |
|  |  |  |  |  |  |  |
| 22 | Was an appropriate method used to ask professionals whether each item is relevant for the construct of interest? | Widely recognized or well justified method used | Only quantitative (survey) method(s) used or assumable that the method was appropriate but not clearly described | Not clear if professionals were asked whether each item is relevant or doubtful whether the method was appropriate | Method used not appropriate or professionals not asked about the relevance of all items |  |
| 23 | Were professionals from all relevant disciplines included? | Professionals from all required disciplines were included | Assumable that professionals from all required disciplines were included, but not clearly described | Doubtful whether professionals from all required disciplines were included or relevant professionals were not included |  |  |
| 24 | Was each item tested in an appropriate number of professionals?  For qualitative studies  For quantitative (survey) studies | ≥7  ≥50 | 4-6  ≥30 | <4 or not clear  <30 or not clear |  |  |
| *Analyses* | |  |  |  |  |  |
|  | |  |  |  |  |  |
| 25 | Was an appropriate approach used to analyse the data? | A widely recognized or well justified approach was used | Assumable that the approach was appropriate, but not clearly described | Not clear what approach was used or doubtful whether the approach was appropriate | Approach not appropriate |  |
| 26 | Were at least two researchers involved in the analysis? | At least two researchers involved in the analysis | Assumable that at least two researchers were involved in the analysis, but not clearly described | Not clear if two researchers were included in the analysis or only one researcher involved in the analysis |  |  |

| **2e. Asking professionals about comprehensiveness** | | | | | | | | |
| --- | --- | --- | --- | --- | --- | --- | --- | --- |
| *Design requirement* | | | **very good** | **adequate** | | **doubtful** | **inadequate** | **NA** |
|  | |  | | |  |  |  |  |
| 27 | Was an appropriate method used for assessing the comprehensiveness of the PROM? | Widely recognized or well justified method used | | | Only quantitative (survey) method(s) used or assumable that the method was appropriate but not clearly described | Doubtful whether the method was appropriate | Method used not appropriate |  |
| 28 | Were professionals from all relevant disciplines included? | Professionals from all required disciplines were included | | | Assumable that professionals from all required disciplines were included, but not clearly described | Doubtful whether professionals from all required disciplines were included or relevant professionals were not included |  |  |
| 29 | Was each item tested in an appropriate number of professionals?  For qualitative studies  For quantitative (survey) studies | ≥7  ≥50 | | | 4-6  ≥30 | <4 or not clear  <30 or not clear |  |  |
| *Analyses* | |  | | |  |  |  |  |
|  | |  | | |  |  |  |  |
| 30 | Was an appropriate approach used to analyse the data? | A widely recognized or well justified approach was used | | | Assumable that the approach was appropriate, but not clearly described | Not clear what approach was used or doubtful whether the approach was appropriate | Approach not appropriate |  |
| 31 | Were at least two researchers involved in the analysis? | At least two researchers involved in the analysis | | | Assumable that at least two researchers were involved in the analysis, but not clearly described | Not clear if two researchers were included in the analysis or only one researcher involved in the analysis |  |  |

| **Box 3. Structural validity** | | | | | | | | | | |  |
| --- | --- | --- | --- | --- | --- | --- | --- | --- | --- | --- | --- |
|  | |  | | | | | | | | |  |
| Does the scale consist of effect indicators, i.e. is it based on a reflective model? yes / no ^1^ | | | | | | | | | | |  |
|  | |  | | | | | | | | |  |
| Does the study concern unidimensionality or structural validity? ^2^ unidimensionality / structural validity | | | | | | | | | | |  |
| *Statistical methods* | | | **very good** | **adequate** | | **doubtful** | | | **inadequate** | | **NA** |
|  |  | |  |  | | |  |  | |  | |
| 1 | | For CTT: Was exploratory or confirmatory factor analysis performed? | Confirmatory factor analysis performed | Exploratory factor analysis performed | | |  | | No exploratory or confirmatory factor analysis performed | | Not applicable |
| 2 | | For IRT/Rasch: does the chosen model fit to the research question? | Chosen model fits well to the research question | Assumable that the chosen model fits well to the research question | | | Doubtful if the chosen model fits well to the research question | | Chosen model does not fit to the research question | | Not applicable |
| 3 | | Was the sample size included in the analysis adequate? | FA: 7 times the number of items and ≥100  Rasch/1PL models: ≥ 200 subjects  2PL parametric IRT models OR Mokken scale analysis: ≥ 1000 subjects | FA: at least 5 times the number of items and ≥100; OR at least 6 times number of items but <100  Rasch/1PL models: 100-199 subjects  2PL parametric IRT models OR Mokken scale analysis: 500-999 subjects | FA: 5 times the number of items but <100  Rasch/1PL models: 50-99 subjects  2PL parametric IRT models OR Mokken scale analysis: 250-499 subjects | | | | FA: < 5 times the number of items  Rasch/1PL models: < 50 subjects  2PL parametric IRT models OR Mokken scale analysis: < 250 subjects | |  |

| *Other* | | |  | |  | |  | |  | |  |
| --- | --- | --- | --- | --- | --- | --- | --- | --- | --- | --- | --- |
|  |  | |  |  | |  | |  | |  | |
| 4 | | Were there any other important flaws in the design or statistical methods of the study? | No other important methodological flaws | |  | | Other minor methodological flaws (e.g. rotation method not described) | | Other important methodological flaws (e.g. inappropriate rotation method) | |  |

^1^ If the scale is not based on a reflective model, unidimensionality or structural validity is not relevant. In this case, the study can be ignored.

^2^ In a systematic review, it is helpful to make a distinction between studies where factor analysis is performed on each (sub)scale separately to evaluate whether the (sub)scales are unidimensional (unidimensionality studies) and studies where factor analysis is performed on all items of an instrument to evaluate the (expected) number of subscales in the instrument and the clustering of items within subscales (structural validity studies).

| **Box 4. Internal consistency** | | | | | |  |
| --- | --- | --- | --- | --- | --- | --- |
|  |  | | | | |  |
| Does the scale consist of effect indicators, i.e. is it based on a reflective model? yes / no ^1^ | | | | | |  |
|  |  | | | | |  |
| *Design requirements* | | **very good** | **adequate** | **doubtful** | **inadequate** | **NA** |
|  |  |  |  |  |  |  |
| 1 | Was an internal consistency statistic calculated for each unidimensional scale or subscale separately? | Internal consistency statistic calculated for each unidimensional scale or subscale |  | Unclear whether scale or sub scale is unidimensional | Internal consistency statistic NOT calculated for each unidimensional scale or sub scale |  |
| *Statistical methods* | |  |  |  |  |  |
|  |  |  |  |  |  |  |
| 2 | For continuous scores: Was Cronbach’s alpha or omega calculated? | Cronbach’s alpha, or Omega calculated |  | Only item-total correlations calculated | No Cronbach’s alpha and no item-total correlations calculated | Not applicable |
| 3 | For dichotomous scores: Was Cronbach’s alpha or KR-20 calculated? | Cronbach’s alpha or KR-20 calculated |  | Only item-total correlations calculated | No Cronbach’s alpha or KR-20 and no item-total correlations calculated | Not applicable |
| 4 | For IRT-based scores: Was standard error of the theta (SE (θ)) or reliability coefficient of estimated latent trait value (index of (subject or item) separation) calculated? | SE(θ) or reliability coefficient calculated |  |  | SE(θ) or reliability coefficient NOT calculated | Not applicable |
|  |  |  |  |  |  |  |
| *Other* | |  |  |  |  |  |
|  |  |  |  |  |  |  |
| 5 | Were there any other important flaws in the design or statistical methods of the study? | No other important methodological flaws |  | Other minor methodological flaws | Other important methodological flaws |  |

^1^ If the scale is not based on a reflective model, internal consistency is not relevant. In this case, the study can be ignored.

| **Box 5. Cross-cultural validity\Measurement invariance** | | | | | |  |
| --- | --- | --- | --- | --- | --- | --- |
| *Design requirements* | | **very good** | **adequate** | **doubtful** | **inadequate** | **NA** |
|  |  |  |  |  |  |  |
| 1 | Were the samples similar for relevant characteristics except for the group variable? | Evidence provided that samples were similar for relevant characteristics except group variable | Stated (but no evidence provided) that samples were similar for relevant characteristics except group variable | Unclear whether samples were similar for relevant characteristics except group variable | Samples were NOT similar for relevant characteristics except group variable |  |
| *Statistical methods* | |  |  |  |  |  |
|  |  |  |  |  |  |  |
| 2 | Was an appropriate approach used to analyse the data? | A widely recognized or well justified approach was used | Assumable that the approach was appropriate, but not clearly described | Not clear what approach was used or doubtful whether the approach was appropriate | Approach not appropriate | Not applicable |
| 3 | Was the sample size included in the analysis adequate? | Regression analyses or IRT/Rasch based analyses: 200 subjects per group  MGCFA*: 7 times the number of items and ≥100 | 150 subjects per group  5 times the number of items and ≥100; OR 5-7 times the number of items but <100 | 100 subjects per group  5 times the number of items but <100 | < 100 subjects per group  <5 times the number of items |  |
| *Other* | |  |  |  |  |  |
| 4 | Were there any other important flaws in the design or statistical methods of the study? | No other important methodological flaws |  | Other minor methodological flaws | Other important methodological flaws |  |

**MGCFA: multi-group confirmatory factor analyses*

| **Box 6. Reliability** | | | | | | | | | | |  | |
| --- | --- | --- | --- | --- | --- | --- | --- | --- | --- | --- | --- | --- |
| *Design requirements* | | | **very good** | | | **adequate** | | **doubtful** | | **inadequate** | **NA** | |
|  |  | |  | |  | |  | |  | | |  |
| 1 | | Were patients stable in the interim period on the construct to be measured? | Evidence provided that patients were stable | Assumable that patients were stable | | | | Unclear if patients were stable | | Patients were NOT stable |  | |
| 2 | | Was the time interval appropriate? | Time interval appropriate |  | | | | Doubtful whether time interval was appropriate or time interval was not stated | | Time interval NOT appropriate |  | |
| 3 | | Were the test conditions similar for the measurements? e.g. type of administration, environment, instructions | Test conditions were similar (evidence provided) | Assumable that test conditions were similar | | | | Unclear if test conditions were similar | | Test conditions were NOT similar |  | |
| *Statistical methods* | | |  |  | | | |  | |  |  | |
| 4 | | For continuous scores: Was an intraclass correlation coefficient (ICC) calculated? | ICC calculated and model or formula of the ICC is described | ICC calculated but model or formula of the ICC not described or not optimal.  Pearson or Spearman correlation coefficient calculated with evidence provided that no systematic change has occurred | | | | Pearson or Spearman correlation coefficient calculated WITHOUT evidence provided that no systematic change has occurred or WITH evidence that systematic change has occurred | | No ICC or Pearson or Spearman correlations calculated | Not applicable | |
| 5 | | For dichotomous/nominal/ordinal scores: Was kappa calculated? | Kappa calculated |  | | | |  | | No kappa calculated | Not applicable | |
| 6 | | For ordinal scores: Was a weighted kappa calculated? | Weighted Kappa calculated |  | | | | Unweighted Kappa calculated or not described | |  | Not applicable | |
| 7 | | For ordinal scores: Was the weighting scheme described? e.g. linear, quadratic | Weighting scheme described | Weighting scheme NOT described | | | |  | |  | Not applicable | |
| *Other* | | |  |  | | | |  | |  |  | |
| 8 | | Were there any other important flaws in the design or statistical methods of the study? | No other important methodological flaws |  | | | | Other minor methodological flaws | | Other important methodological flaws |  | |

| **Box 7. Measurement error** | | | | | | | | | |  | |
| --- | --- | --- | --- | --- | --- | --- | --- | --- | --- | --- | --- |
| *Design requirements* | | | **very good** | | **adequate** | | **doubtful** | | **Inadequate** | **NA** | |
|  |  | |  |  | |  | |  | | |  |
| 1 | | Were patients stable in the interim period on the construct to be measured? | Patients were stable (evidence provided) | | Assumable that patients were stable | | Unclear if patients were stable | | Patients were NOT stable |  | |
| 2 | | Was the time interval appropriate? | Time interval appropriate | |  | | Doubtful whether time interval was appropriate or time interval was not stated | | Time interval NOT appropriate |  | |
| 3 | | Were the test conditions similar for the measurements? (e.g. type of administration, environment, instructions) | Test conditions were similar (evidence provided) | | Assumable that test conditions were similar | | Unclear if test conditions were similar | | Test conditions were NOT similar |  | |
| *Statistical methods* | | |  | |  | |  | |  |  | |
|  | |  |  | |  | |  | |  |  | |
| 4 | | For continuous scores: Was the Standard Error of Measurement (SEM), Smallest Detectable Change (SDC) or Limits of Agreement (LoA) calculated? | SEM, SDC, or LoA calculated | | Possible to calculate LoA from the data presented | |  | | SEM calculated based on Cronbach’s alpha, or on SD from another population | Not applicable | |
| 5 | | For dichotomous/nominal/ordinal scores: Was the percentage (positive and negative) agreement calculated? | % positive and negative agreement calculated | | % agreement calculated | |  | | % agreement not calculated | Not applicable | |
| *Other* | | |  | |  | |  | |  |  | |
| 6 | | Were there any other important flaws in the design or statistical methods of the study? | No other important methodological flaws | |  | | Other minor methodological flaws | | Other important methodological flaws |  | |

| **Box 8. Criterion validity** | | | | | |  |
| --- | --- | --- | --- | --- | --- | --- |
|  | | **very good** | **adequate** | **doubtful** | **inadequate** | **NA** |
| *Statistical methods* | |  |  |  |  |  |
|  |  |  |  |  |  |  |
| 1 | For continuous scores: Were correlations, or the area under the receiver operating curve calculated? | Correlations or AUC calculated |  |  | Correlations or AUC NOT calculated | Not applicable |
| 2 | For dichotomous scores: Were sensitivity and specificity determined? | Sensitivity and specificity calculated |  |  | Sensitivity and specificity NOT calculated | Not applicable |
| *Other* | |  |  |  |  |  |
|  |  |  |  |  |  |  |
| 3 | Were there any other important flaws in the design or statistical methods of the study? | No other important methodological flaws |  | Other minor methodological flaws | Other important methodological flaws |  |

| **Box 9. Hypotheses testing for construct validity** | | | | | |  |
| --- | --- | --- | --- | --- | --- | --- |
|  |  |  |  |  |  |  |
| **9a. Comparison with other outcome measurement instruments (convergent validity)** | | | | | |  |
|  |  |  |  |  |  |  |
| *Design requirements* | | **very good** | **adequate** | **doubtful** | **inadequate** | **NA** |
|  |  |  |  |  |  |  |
| 1 | Is it clear what the comparator instrument(s) measure(s)? | Constructs measured by the comparator instrument(s) is clear |  |  | Constructs measured by the comparator instrument(s) is not clear |  |
| 2 | Were the measurement properties of the comparator instrument(s) sufficient? | Sufficient measurement properties of the comparator instrument(s) in a population similar to the study population | Sufficient measurement properties of the comparator instrument(s) but not sure if these apply to the study population | Some information on measurement properties of the comparator instrument(s) in any study population | No information on the measurement properties of the comparator instrument(s), OR evidence for insufficient measurement properties of the comparator instrument(s) |  |
| *Statistical methods* | |  |  |  |  |  |
| 3 | Was the statistical method appriopriate for the hypotheses to be tested? | Statistical method was appropriate | Assumable that statistical method was appropriate | Statistical method applied NOT optimal | Statistical method applied NOT appropriate |  |

| *Other* | |  |  |  |  |  |
| --- | --- | --- | --- | --- | --- | --- |
|  |  |  |  |  |  |  |
| 4 | Were there any other important flaws in the design or statistical methods of the study? | No other important methodological flaws |  | Other minor methodological flaws (e.g. only data presented on a comparison with an instrument that measures another construct) | Other important methodological flaws |  |

| **9b. Comparison between subgroups (discriminative or known-groups validity)** | | | | | |  |
| --- | --- | --- | --- | --- | --- | --- |
|  |  | | | | |  |
| *Design requirements* | | **very good** | **adequate** | **doubtful** | **inadequate** | **NA** |
|  |  |  |  |  |  |  |
| 5 | Was an adequate description provided of important characteristics of the subgroups? | Adequate description of the important characteristics of the subgroups | Adequate description of most of the important characteristics of the subgroups | Poor of no description of the important characteristics of the subgroups |  |  |
| *Statistical methods* | |  |  |  |  |  |
|  |  |  |  |  |  |  |
| 6 | Was the statistical method appropriate for the hypotheses to be tested? | Statistical method was appropriate | Assumable that statistical method was appropriate | Statistical method applied NOT optimal | Statistical method applied NOT appropriate |  |
| *Other* | |  |  |  |  |  |
|  |  |  |  |  |  |  |
| 7 | Were there any other important flaws in the design or statistical methods of the study? | No other important methodological flaws |  | Other minor methodological flaws (e.g. only data presented on a comparison with an instrument that measures another construct) | Other important methodological flaws |  |

| **Box 10. Responsiveness** | | | | | |  |
| --- | --- | --- | --- | --- | --- | --- |
|  |  |  |  |  |  |  |
| **10a. Criterion approach (i.e. comparison to a gold standard)** | |  |  |  |  |  |
|  |  |  |  |  |  |  |
|  | | **very good** | **adequate** | **doubtful** | **inadequate** | **NA** |
| *Statistical methods* | |  |  |  |  |  |
|  |  |  |  |  |  |  |
| 1 | For continuous scores: Were correlations between change scores, or the area under the Receiver Operator Curve (ROC) curve calculated? | Correlations or Area under the ROC Curve (AUC) calculated |  |  | Correlations or AUC NOT calculated | Not applicable |
| 2 | For dichotomous scales: Were sensitivity and specificity (changed versus not changed) determined? | Sensitivity and specificity calculated |  |  | Sensitivity and specificity NOT calculated | Not applicable |
| *Other* | |  |  |  |  |  |
|  |  |  |  |  |  |  |
| 3 | Were there any other important flaws in the design or statistical methods of the study? | No other important methodological flaws |  | Other minor methodological flaws | Other important methodological flaws |  |

| **10b. Construct approach (i.e. hypotheses testing; comparison with other outcome measurement instruments)** | | | | | | |
| --- | --- | --- | --- | --- | --- | --- |
|  |  |  |  |  |  |  |
| *Design requirements* | | **very good** | **adequate** | **doubtful** | **inadequate** | **NA** |
|  |  |  |  |  |  |  |
| 4 | Is it clear what the comparator instrument(s) measure(s)? | Constructs measured by the comparator instrument(s) is clear |  |  | Constructs measured by the comparator instrument(s) is not clear |  |
| 5 | Were the measurement properties of the comparator instrument(s) sufficient? | Sufficient measurement properties of the comparator instrument(s) in a population similar to the study population | Sufficient measurement properties of the comparator instrument(s) but not sure if these apply to the study population | Some information on measurement properties of the comparator instrument(s) in any study population | NO information on the measurement properties of the comparator instrument(s) OR evidence of poor quality of comparator instrument(s) |  |
| *Statistical methods* | |  |  |  |  |  |
|  |  |  |  |  |  |  |
| 6 | Was the statistical method appropriate for the hypotheses to be tested? | Statistical method was appropriate | Assumable that statistical method were appropriate | Statistical method applied NOT optimal | Statistical method applied NOT appropriate |  |
| *Other* | |  |  |  |  |  |
|  |  |  |  |  |  |  |
| 7 | Were there any other important flaws in the design or statistical methods of the study? | No other important methodological flaws |  | Other minor methodological flaws | Other important methodological flaws |  |

| **10c. Construct approach: (i.e. hypotheses testing: comparison between subgroups)** | | | | | | |
| --- | --- | --- | --- | --- | --- | --- |
|  |  | | | | |  |
| *Design requirements* | | **very good** | **adequate** | **doubtful** | **inadequate** | **NA** |
|  | |  |  |  |  |  |
| 8 | Was an adequate description provided of important characteristics of the subgroups? | Adequate description of the important characteristics of the subgroups | Adequate description of most of the important characteristics of the subgroups | Poor or no description of the important characteristics of the subgroups |  |  |
| *Statistical methods* | |  |  |  |  |  |
|  |  |  |  |  |  |  |
| 9 | Was the statistical method appropriate for the hypotheses to be tested? | Statistical method was appropriate | Assumable that statistical method was appropriate | Statistical method applied NOT optimal | Statistical method applied NOT appropriate |  |
| *Other* | |  |  |  |  |  |
|  |  |  |  |  |  |  |
| 10 | Were there any other important flaws in the design or statistical methods of the study? | No other important methodological flaws |  | Other minor methodological flaws | Other important methodological flaws |  |

| **10d. Construct approach: (i.e. hypotheses testing: before and after intervention)** | | | | | | |
| --- | --- | --- | --- | --- | --- | --- |
|  |  | | | | |  |
| *Design requirements* | | **very good** | **adequate** | **doubtful** | **inadequate** | **NA** |
|  |  |  |  |  |  |  |
| 11 | Was an adequate description provided of the intervention given? | Adequate description of the intervention |  | Poor description of the intervention | NO description of the intervention |  |
| *Statistical methods* | |  |  |  |  |  |
|  |  |  |  |  |  |  |
| 12 | Was the statistical method appropriate for the hypotheses to be tested? | Statistical method was appropriate | Assumable that statistical method was appropriate | Statistical method applied NOT optimal | Statistical method applied NOT appropriate |  |
| *Other* | |  |  |  |  |  |
|  |  |  |  |  |  |  |
| 13 | Were there any other important flaws in the design or statistical methods of the study? | No other important methodological flaws |  | Other minor methodological flaws | Other important methodological flaws |  |
